# The diffusion model visualizer: an interactive tool to understand the diffusion model parameters

**DOI:** 10.1007/s00426-018-1112-6

**Published:** 2018-10-25

**Authors:** Rainer W. Alexandrowicz

**Affiliations:** Alpen-Adria-Universitat Klagenfurt, Klagenfurt, Austria

**Keywords:** Reaction time analysis, Data visualization, Diffusion model

## Abstract

Response time (RT) data play an important role in psychology. The diffusion model (DM) allows to analyze RT-data in a two-alternative-force-choice paradigm using a particle drift diffusion modeling approach. It accounts for right-skewed distributions in a natural way. However, the model incorporates seven parameters, the roles of which are difficult to comprehend from the model equation. Therefore, the present article introduces the diffusion model visualizer (DMV) allowing for interactive manipulation of each parameter and plotting the resulting RT densities. Thus, the DMV serves as a valuable tool for understanding the specific role of each model parameter. It may come in handy for didactical purposes and in research context. It allows for tracking down parameter estimation problems by delivering the model-based ideal densities, which can be juxtaposed to the data-based densities. It will also serve a valuable purpose in detecting outliers. The article describes the basics of the DM along with technical details of the DMV and gives several hints for its usage.

## Introduction

Response times (RTs) for decisions constitute a valuable source of information in psychological research. They occur in simple reaction tasks (e.g., response to the occurence of a stimulus such as a light) and recognition tasks (go/no-go tasks and choice tasks, responding to stimuli with certain characteristics, but not to distractors lacking these characteristics). In the present context, we will focus primarily on data from recognition tasks.

RT distributions are right-skewed by nature, requiring appropriate handling when applying analysis techniques assuming normally distributed data. Because of the technical efforts required to record RT data, we usually use experimental designs to generate response data. These are primarily evaluated with ANOVA-based methods, which do assume normal distributions. As a remedy, several ways of handling the skewed data have been proposed: some argue that the ANOVA *F* test is sufficiently robust against non-normality and, therefore, do not correct at all (e.g., Hays, 1994, p. 406). Others apply transformations to approach normal distribution, like the Box–Cox transformation (e.g., Seber & Lee, 2003, ch. 10.3.2), the square root transformation or, more general, the family of power transformations (e.g., Cohen, Cohen, West & Aiken, 2003, p. 233 and p. 245), the logarithmic transformation (Cohen et al., 2003, p. 245), rank-based normalization (Cohen et al., 2003, p. 247), the shifted power transformation (e.g., Atkinson, 1985, p. 184), and others (e.g., Gro, 2004, ch. 6). A third line of handling is elimination of the values considered as outliers, i.e., both fast and slow responses (e.g., Voss, Nagler & Lerche, 2016). Fourth, one may as well try to apply other models than the normal one, as did Matzke and Wagenmakers (2009) by probing the ex-Gaussian and the shifted-Wald distribution, however, with limited success. Although some researchers found parameters of descriptive parameterizations of RT distributions to be useful (e.g., Schmiedek, Oberauer, Wilhelm, Sü & Wittmann, 2007; Spieler, Balota & Faust, 1996), the primary point of criticism concerning shifted Wald and ex-Gaussian parameterization is probably that they are not motivated by a psychological theory. Another shortcoming is that they neglect the information in classification errors. Consequently, they are not suited to cope with speed–accuracy trade-offs.

In contrast, the diffusion model constitutes an entirely different approach by drawing on particle diffusion theory. This approach offers a compelling principle to describe the skewed RT distributions typically resulting from human decision formation. However, due to its complexity (involving seven model parameters, as will be demonstrated below), it is hard to conceive, how the various parameters affect the resulting RT density curves. Therefore, the present article introduces a visualization tool allowing for exactly tracking and scrutinizing the role of each parameter and their interplay in great detail. The text is structured as follows: after a short introduction of the diffusion model (including numerous hints to relevant sources), the visualization tool is presented along with some technical details. Finally, we will discuss useful applications of the visualization program.

## The diffusion modeling approach to RT analysis

The diffusion model (DM; Ratcliff, 1978, 2013), also termed Ratcliff DM, drift diffusion model (DDM, e.g., Bogacz, Brown, Moehlis, Holmes & Cohen, 2006; Correll, Wittenbrink, Crawford & Sadler, 2015; Dutilh et al., 2016, subm.), or Wiener diffusion model with absorbing boundaries (e.g., Grasman, Wagenmakers & van der Maas, 2009) takes into account both RT and accuracy of speeded binary decisions like those occurring in a two-alternative-forced-choice (2AFC or TAFC) paradigm. Respondents have to select one out of two response alternatives, possibly under time pressure, while RT and correctness are recorded for each decision. The simultaneous availability of both provides a means for disentangling the speed–accuracy trade-off dilemma (for a detailed account see Heitz, 2014 or Ratcliff, 1978, pp. 93–97). How DM analyses improve our understanding of relatively fast decisions has been a major topic in two recent overview articles (Forstmann, Ratcliff & Wagenmakers, 2016; Ratcliff, Smith, Brown & McKoon, 2016).

### The concept of the diffusion model

Basically, the model assumes that cognitive information accumulation and processing takes place in form of a sequential sampling process. After stimulus presentation, the respondent collects, processes, and accumulates stimulus features, which favor either decision A or decision B. The model assumes that this accumulation corresponds to neural activity in some way without making further assumptions regarding details of the process. Several studies collected empirical evidence supporting the plausibility of this assumption (e.g., Gold & Shadlen, 2007, Forstmann et al., 2016, Heekeren, Marrett, Bandettini & Ungerleider, 2004; Heekeren, Marrett & Ungerleider, 2008; Ho, Brown & Serences, 2009; Lo & Wang, 2006; Ma, Beck & Pouget, 2008; Soltani & Wang, 2010).

We conceive of the decision process as a random walk (i.e., discrete), but with the sampling assumed so fast that it can be expressed by a Wiener (i.e., a continuous-time) diffusion process, with a large noise component (cf. Navarro & Fuss, 2009). The two resulting RT distributions form First-Passage Time distributions (FPT; e.g., Feller, 1968, ch. XIV.6 or Feller, 1971, ch. XIV.5), which can be traced back to what Siegmund (1986) has termed the “grandfather of all such problems” (p. 361), the one sample Kolmogorov–Smirnov statistic. Cox and Miller (1965) provide a fundamental treatise regarding Brownian motion and absorption.

Technically, we deal with sequential sampling models in the sense of Wald (1945, 1947), assuming here that a decision is the result of (noisy) evidence accumulation across a period of time eventually passing a critical threshold. Laming (1968) presents a series of experiments specifically linking the decision process to the random walk model. However, in contrast to random walk models, the DM assumes both, evidence and time, to be continuous. The DM is therefore frequently described as a special case of the more general class of sequential sampling models, which are characterized by sampling of relative evidence, and contrasted to the class of Accumulator Models characterized by absolute evidence criteria (Bogacz et al., 2006; Ratcliff et al., 2016).

### The four main parameters of the DM

The “classical” DM as formulated by Ratcliff (1978) employs four model parameters, which can be perceived as the “main” parameters carrying the fundamental meaning of the model in substantive terms (extensions will be discussed in “Parameter variability/variability parameters”).

**Fig. 1:**
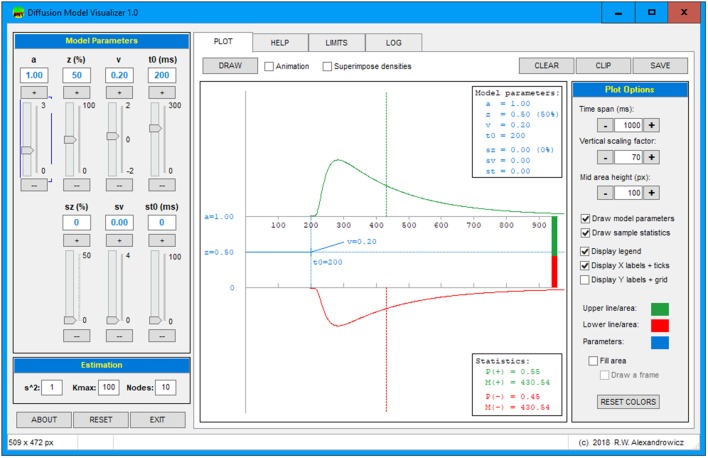
Graphical user interface of the diffusion model visualizer showing an example of a distribution. Notes: The horizontal axis represents the RT. The top section (green curve) shows the RT density for positive responses and the bottom section (red curve) the RT density for negative responses. For reaction times shorter than $t_0$, the probability of a response is zero. In the left panel there are seven sliders allowing for adjusting the four main (upper row) and the three variability parameters (lower row). Below, we find entry fields for parameters affecting the computation of the density curves. The middle section contains the plot: The vertical dashed lines indicate the expected means and the stacked bars at the end of the horizontal axis show the expected proportions of positive and negative responses. The shaded blue bars represent the variability parameters $s_z$, $s_{t_0}$, and $s_\nu$. In the upper and lower right corner, the chosen parameter values (blue) and some descriptive statistics (red) are printed. At right hand side, we find the options to fine-tune the appearance of the diagram

Figure 1 shows an illustration of the RT densities of both reaction alternatives along with the four main model parameters. The parameter *a* is the upper threshold of accumulated evidence in favor of decision alternative A required to issue the respective response (usually pressing a key). The lower boundary is set to a value of zero, hence *a* is the boundary separation and reflects the threshold difference at the same time. The parameter *z* denotes the location of the starting point. It can be expressed as an absolute value on the same scale as *a*, then $0< z < a$; however, it has proven convenient to rescale it to represent the starting position relative to *a*, then $0< z < 1$ (which will be adopted here). The parameter $t_0$ (or $T_{\mathrm {ER}}$) collects all time components not related to decision-making but for encoding and response. The parameter $\nu$ denotes the drift rate of information accumulation, which is the average amount of evidence gathered per time slice. It can take positive and negative values.

### Parameter interpretation from a psychological point of view

While the model parameters convey a compelling interpretation from a theoretical point of view, we have to provide empirical evidence corroborating these theoretical assumptions from a substantive (psychological) perspective. The following selection of results regarding parameter validation illustrates that we already dispose of ample evidence supporting the theoretical view. For studies systematically exploring parameter validity see, for example, Arnold, Broder and Bayen (2015), Lerche and Voss (2017), Ratcliff and Rouder (1998), Voss, Rothermund and Voss (2004), or Wagenmakers, Ratcliff, Gomez and McKoon (2008).

#### Boundary separation *a*

Large values of *a* are assumed to indicate the subject’s response caution, which is under subjective control and determined prior to the start of each trial (e.g., Wagenmakers, 2009). In this sense, Voss et al. (2004) termed this parameter the “response criterion”. Ratcliff and Rouder (1998) established a speed vs. accuracy condition by instructing the respondents to respond as quickly as possible in the first case or to decide as accurate as possible in the latter case. They found marked differences in the *a* parameter estimates between the two conditions. Also Voss et al. (2004) found significantly increased values of this parameter when instructing respondents to “work especially carefully and to avoid mistakes” (p. 1211). Likewise, van Ravanzwaaij and Oberauer (2009) found the speed-accuracy instruction to affect primarily *a*. Arnold et al. (2015) varied the speed-accuracy instruction by giving negative feedback either when the response was wrong (accuracy condition) or when the response took more than 1000 ms (speed condition), yielding the expected differences in *a* as well. Moreover, the boundary separation also seems to increase with age. Ratcliff and McKoon (2008) quote a large series of studies indicating that this effect is due to increased conservatism of older adults (p. 911).

#### Response bias *z*

The starting point (or bias) parameter *z* is understood as a bias of the individual, reflecting the a priori expectation, whether the next stimulus will be a positive or negative example (i.e., which response will likely be the adequate one). Accordingly, Ratcliff and McKoon (2008) found variations in *z* in relation to the proportion of left- and right-moving stimuli (which the subjects were told in advance); Arnold et al. (2015) also found variations in *z* by varying the proportion of old and new items in a recognition memory experiment; Voss et al. (2004) further revealed that the starting point varies when one of the two responses is offered a reward.

#### Drift parameter ${\nu }$

Ratcliff and McKoon (2008) describe the drift parameter as the “quality, or strength, of the information available from a stimulus” (p. 901). Hence, large (absolute) values indicate that the stimulus allows for a fast decision, while values close to zero indicate that the decision might rather rest upon guessing. Ratcliff (1978) considered the drift parameter “to alone represent input from memory into the decision system” (p. 70). However, in a between-subject comparison of identical tasks, $\nu$ may as well be seen as the parameter representing “perceptual sensitivity” (Voss et al. 2004, p. 1208). This interpretation is rather appealing, as it allows for modeling a subject’s information processing speed independent of speed–accuracy preference or conservatism (which is covered by *a*) or motor response-execution speed (covered by $t_0$). It is further in line with Schmiedek et al. (2007), who found a relation of the drift parameter to working memory. Also, van Ravenzwaaij, Brown and Wagenmakers (2011) relate individual differences in general intelligence to the drift parameter and Ranger, Kuhn and Szardenings (2016) characterize $\nu$ generally as “(...) the subject’s capability to process information.” (p. 124).

Voss et al. (2004) found the drift rate to vary corresponding to the increased difficulty of the task. Also Ratcliff and McKoon (2008) found that stimuli of varying difficulty affected exclusively the drift parameter. Similarly, van Ravanzwaaij and Oberauer (2009) found $\nu$ to correspond to the stimulus–response compatibility (p. 469). Arnold et al. (2015) presented some of the old stimuli of a recognition experiment once and some of them twice along with new items, yielding significant differences in $\nu$ across these three conditions.

#### Non-decision time component ${t}_{{0}}$

The $t_0$ parameter comprises all processes not involved in decision-making. These embrace encoding of the stimulus, response preparation, and motor response. Voss et al. (2004) induced a response handicap condition, in which respondents were instructed to use one and the same finger for all keyboard responses (“C” and “M”), requiring them to press the “B”-key with the same finger to start the trial. They found on average a significantly increased value for the $t_0$-parameter (compared to a standard experimental condition, not involving this response handicap), suggesting that the non-decision parameter actually reflects the motoric complexity of a task. The same conclusion was achieved by Gomez et al. (2015), who by varying the response modality (eye movement, key pressing, and pointing on a touchscreen; p. 1518) found these three modalities to affect only $t_0$ (and its variability, $s_{t_0}$), but none of the other model parameters. Lerche and Voss (2017) required their respondents to press the response key not once (as usual), but three times in a row, after coming to a decision. They found clear effects upon the $t_0$-parameter, thus corroborating the validity of this parameter.

### The model equations

The DM relies on two sources of information, the (non-)matching response rate and the two RT distributions. Resorting to the “gamblers ruin problem” (or “classical ruin problem”, as denoted by Feller 1968, p. 342), which is of discrete nature, Ratcliff (1978) restated winning (or losing) a dollar as the (non-)matching of the features of a probe item and a memory item. By considering information accumulation as a continuous process, he arrives at

1 $$\begin{aligned} P(-|a,z,\nu ) = \frac{\mathrm {e}^{-(2\nu {}a/s^2)} - \mathrm {e}^{-(2\nu {}z/s^2)}}{\,\mathrm {e}^{-(2\nu {}a/s^2)} - 1 \;\;\;\;\;\;\;\;\;\;\;\,}, \end{aligned}$$

for the probability of a non-matching response and the finishing time density for negative responses is given as

2 $$\begin{aligned} g_{-}(t,a,z,\nu ) = \frac{\pi {}s^2}{a^2} \mathrm {e}^{-\frac{z\nu }{s^2}} \sum _{k=1}^{\infty } k\sin \left( \frac{\pi {}zk}{a} \right) \mathrm {e}^{-\frac{1}{2}\left( \frac{\nu ^2}{s^2}+\frac{\pi ^2k^2s^2}{a^2}\right) t} \end{aligned}$$

(p. 70, Equations (A8) and (A9), notation adapted). The probability of and density for positive responses is obtained analoguously by applying $\nu _+ = -\nu$ and $z_+ = a-z$. The term $s^2$ denotes the variance of the Brownian motion within one trial (therefore termed intra-trial variability of the drift), which is not a model parameter, but rather a constant. It has to be set to an appropriate value prior to parameter estimation to make the model identified (cf. Ratcliff et al., 2016, p. 262). The choice of *s* is not critical, as it regards only the scale of the estimated parameters; two values are often observed, $s=1$ and $s=0.1$.

Equation (2) has been previously published, e.g., in Feller (1968, eq. 6.15), who also notes that it is known as “Fürth’s formula for first passages” in physical diffusion theory (Feller, 1968, p. 359). Busemeyer and Diederich (2010, esp. Appendix to ch. 4) provide a comprehensible derivation of the model equations. Ratcliff (1978) also showed an alternative means to derive the first-passage time distribution (2) as the solution of a partial differential equation (PDE, also known as Fokker–Planck Backward Equation in statistical mechanics), thus circumventing the approximation of the infinite sum involved in Eq. (2).

### Parameter variability/variability parameters

For applications it is reasonable to take further sources of variability into account and to provide for the according parameters. The core aspect of parameter variability is that it seems unplausible to assume respondents’ attention to remain constant throughout the entire experiment. Rather, we have to expect a trial-to-trial-fluctuation of the model parameters.

The variability of the drift parameter $s_\nu$ (Ratcliff, 1978 and other authors use $\xi$ for the mean and $\eta$ for the standard deviation of $\nu$) has always been an integral element of the drift diffusion model. It allows to account for variability in encoding in memory, but also predicts slower RTs for incorrect responses than for correct ones (cf. Ratcliff & Rouder, 1998, p. 348; Ratcliff et al., 2016, p. 267).

Ratcliff and Rouder (1998) introduced further the starting point variability parameter $s_z$ to explain error responses “slower than correct responses at intermediate levels of accuracy” and “faster than correct responses at extreme levels of accuracy” (p. 349). Moreover, Ratcliff and Tuerlinckx (2002) introduced also a variability parameter of the encoding and reaction time, $s_{t_0}$ to model large variability of very fast responses (esp. the 0.1 quantile of the RT distribution, see ibid., p. 441).

#### Distributional assumptions and estimation

The drift rate variability is assumed to follow a normal distribution with mean $\nu$ and standard deviation $s_\nu$. In contrast, the encoding and reaction time component $t_0$ and the starting point *z* are modelled assuming a uniform distribution, with range $s_{t_0}$ and $s_z$, respectively. Thus, we yield for the effective parameters $\nu ^*$, $z^*$, and $t_0^*$:

3 $$\begin{aligned} \nu ^*&\sim {} N(\nu ,s_\nu ^2), \end{aligned}$$

4 $$\begin{aligned} z^*&\sim {} U(z - s_z/2 ,z + s_z/2), \end{aligned}$$

5 $$\begin{aligned} t_0^*&\sim {} U(t_0 - s_{t_0}/2,t_0 + s_{t_0}/2). \end{aligned}$$

The three variability parameters do not have a psychological interpretation of their own, but rather allow for capturing random disturbances without invalidating the model or deteriorating parameter estimates and model fit. However, some authors argue in favor of fixing the variability parameters, either because this might improve estimability of the four main parameters (e.g., Lerche & Voss, 2016; 2017), or to keep the entire procedure as simple as possible (e.g., van Ravenzwaaij, Donkin & Vanderkerckhove, 2017).

The variability parameters are not part of the model Eqs. (1) and (2), but rather have to be found by numerical integration in the parameter estimation process (see “Behind the scenes: a few technical details”). They have (compared to the four main parameters) little influence upon the resulting density curves, which can easily be checked with the tool presented in the next section.

#### Further extensions

Next to the variability parameters, several model extensions have been proposed, e.g., Voss et al. (2010) introducing a response-execution bias parameter; Krajbich and Rangel (2011) providing for a three-alternative decision design; Diederich (1994) using an Ornstein–Uhlenbeck process with drift; Tuerlinckx and Boeck (2005), van der Maas et al. (2011), and Molenaar, Tuerlinckx and van der Maas (2015) bridging the DM to item response theory (IRT) models, or Vandekerckhove et al. (2011) introducing a hierarchical approach with responses (level 1) nested within respondents (level 2).

## The diffusion model visualizer

The diffusion model has proven to be a valuable tool for evaluating both response correctness and RT in decision-making processes. However, the specific roles the various model parameters play in generating the densities of positive and negative responses are hardly apprehensible from the model equations. For that purpose, the diffusion model visualizer (DMV) has been developed visualizing the RT density curves for a given parameter constellation. Figure 1 shows the graphical user interface (GUI) of this program.

### The DMV GUI

At the left, there is a panel with seven sliders allowing adjustment of each parameter. These sliders can be changed with the mouse, using the “+” and “–” buttons, using the PgUp and PgDown buttons, or by entering values into the respective text fields. Below, three entries allow for setting $s^2$, the number of iterations to approach the infinite sum of Eq. (2), and the number of nodes to be used for the numerical integration required for the variability parameters. The middle section of the GUI shows the resulting density curves along with a symbolic depiction of the actual model parameters. At the right, graphical and computational options allow for fine-tuning the plot. On top of the screen, there are four tabs allowing for switching from the PLOT page to a HELP page with some basic instructions and usage hints, the LIMITS page, which allows for increasing the parameter limits (for experimental purposes), and the LOG page with some numerical details of the calculations. The DRAW button invokes the calculations and delivers the diagram with the currently set options. The CLEAR button empties the canvas, the CLIP button copies the current diagram to the clipboard, and the SAVE button allows for storing the plot to disk. A bold face caption of the DRAW button indicates pending parameter changes.

Next to the DRAW button there is the Animation checkbox. It causes the plot to be drawn instantly upon slider movements, which gives a vivid impression how the curves change with parameter modifications. Next to this option, there is a Superimpose densities checkbox, which helps to compare various parameter constellations.

The Plot options panel allows for adaption of the diagram to specific scenarios (e.g., for a closer inspection of the densities’ ascending tails by decreasing the Time span). The fill area option might prove useful in cases, in which a very small image is to be conveyed, for example in a matrix plot.

### DMV usage

The default parameter setting is $a=1$, $z=0.5$, $\nu =0.2$, and $t_0=300$, with the variability parameters set to zero and $s^2=1$. This configuration results in two fairly similar density curves. Shifting the *a* slider upwards shows how both densities become increasingly flatter the larger the value of *a*. This effect is comprehensible, as a larger *a* indicates more evidence accumulation towards one of the two decision boundaries thus causing elongated reaction times. In contrast, when we decrease *a*, the densities increase for short reaction times changing their shape drastically. Both curves change in a similar way, as positive and negative responses are affected equally from changes in *a*.

Changing the starting point *z* causes a strong dissimilarity of the two curves. Increase of *z* causes the upper curve to grow and the lower curve to shrink and vice versa. This effect is near at hand, as being biased towards, say, the upper decision fosters its choice while much more evidence is required to come to the lower one, and vice versa. At the same time, the two curves also change their shape drastically and in an asymmetrical way, because not only the response probabilities (as given in Eq. 1) change, but also the respective RT mean values.

Changing the $\nu$ parameter also in-/decreases the kurtosis of the two curves, as did *z*. But in contrast to *z*, the shape changes are more symmetrical, less pronounced, and the means will not differ notably.

Finally, moving the $t_0$ slider shifts both densities horizontally. This is evident from the model definition, as the encoding and RT is not associated with the decision process itself thus leaving the densities’ shapes unaltered.

The three variability parameters are initially set to zero. Increasing $s_v$ will cause a slight increase in short reaction times moving the mode of the curve somewhat to the left. The $s_z$ parameter causes a slight increasing of the “peakedness” (i.e., the kurtosis) of both densities. The $s_{t_0}$ parameter “flattens” the ascending tails of the densities a bit and thus reduces their kurtosis. Note that due to the computational burden, the variability parameter sliders will take effect not before releasing the mouse button (in contrast to the four main parameter sliders; see “Behind the scenes: a few technical details”).

To gain an impression of the effect of the various parameter configurations, the Superimpose densities checkbox allows for plotting multiple curves in one plot. Note that this option becomes automatically unchecked, if the Draw model parameters options is checked and the value of *a* is changed. Otherwise, the dislocated baselines (indicating the de-/increasing *a*) would be drawn one over the other, thus rendering the diagram distorted. Alternatively, one could also uncheck the Draw model parameters option, which will leave the two baselines in place thus keeping the diagram intact (this applies only if you want to change *a*; for examining the other parameters, using Superimpose densities and Draw model parameters conjointly will provide highly informative diagrams).

While it is relatively easy to describe the main effects of changing one parameter at a time, the impact of multiple changes is much more complex and is, therefore, left to the reader. As an introductory example, interested readers could increase the (uncommonly low) $\nu$ (which is 0.2 at start-up) to a more frequently observed value of 1 and inspect the changes.

### Behind the scenes: a few technical details

The program implements the model Eqs. (1) and (2). The infinite sum contained in Eq. (2) is approximated by 100 steps. This value can be changed with the Kmax option. However, program testing showed that too small a Kmax (simulations suggest below approximately 50) may cause artifacts (spikes in the vicinity of $t_0$) with certain parameter configurations due to numerical inaccuracies. A preliminary sensitivity analysis revealed that the default value should suffice for most cases (in most cases, the sum converges after much fewer iterations). Readers are, therefore, advised to increase Kmax with caution, as larger values will increase the computational burden considerably, in particular when working with the variability parameters.

The effects of the three variability parameters are induced by integrating over the respective distributions as given by Eqs. (3), (4), and (5) (cf. Ratcliff & Tuerlinckx, 2002, esp. App. B). The integration is implemented using the trapezoidal rule with ten nodes across the respective ranges as given in Eqs. (3), (4), and (5). For $\nu$, the integration interval is $\nu \pm 4s_\nu$. The nodes option allows for changing the number of nodes, but again, use this option with caution, increasing it will also cause considerable computational burden, while decreasing will lead to numerical inaccuracies.

Because the numerical integration routines invoked by the variability parameters are computationally expensive, especially when activating two or all the three of them simultaneously, plotting may slow down. Therefore, the Animation feature regarding the $s_z$-, the $s_\nu$-, and the $s_{t_0}$-sliders only update the plot upon releasing the mouse button, while the *a*-, *z*-, $\nu$-, and $t_0$-sliders invoke the plot update immediately upon mouse movement.

The intra-trial variance of the Brownian motion, $s^2$, defaults to one with an option for change. Changing $s^2$ will alter the plot unless the other parameter values, which are linearly related to $s^2$ [see model Eqs. (1) and (2)], are adapted accordingly.

The DMV is written in Free Pascal/Lazarus (Free Pascal Team 1993–2016; Lazarus Team 1993–2016). This programming environment has three advantages relevant to our endeavour: first, it allows generation a fast executing code, which is necessary as complex calculations are performed. Second, it supports building clearly-arranged GUIs with little effort. Thirdly, it allows for cross-compiling, i.e., generating binaries for a Linux or a Mac environment as well (at the moment, only a Windows version is provided; in case a version for a different platform is required, please contact the author; under Linux, the program can be executed using the wine emulator).

## Applying the DMV

Several applications of the program can be thought of: first of all, it may serve as a valuable tool for educational purposes, allowing for a vivid illustration of how the various parameters affect the resulting density curves. This is an important task, because the diffusion model uses seven parameters whose effects on the polymorphic RT densities and response probabilities are complex and difficult to understand—equally for students and researchers new to the model. The DMV allows exploration of the effect of the various model parameters upon the decision accuracy and the RT distribution, i.e., not only its mean and standard deviation, but also the higher moments skewness and kurtosis—or even the entire shape as such.

The diffusion model differs fundamentally from more frequently applied models in psychology, like the Generalized Linear Model (GLM; McCullagh & Nelder, 1989) including multi-level structures and structural equation modeling extensions (Skrondal & Rabe-Hesketh, 2004), or Item Response Theory models (IRT; de Ayala, 2009). The most important difference is that it is not a member of the exponential family (Barndorff-Nielsen, 1978) thus follows quite a different mathematical structure, viz. differential equations. Moreover, DMs are still rather rarely applied (but showing a strongly increasing tendency), so researchers have so far had fewer opportunities to become familiar with them. While the model equations rather conceal information, the animated illustration allows for grasping the basic concept and dynamics of the model and the roles its parameters play in an intuitive way.

The DMV might also prove useful in research. It could come in handy when parameter estimation problems are observed (e.g., estimates exhibit unexpected values or the estimation routine would not terminate in a regular fashion). Then, one could juxtapose the observed RT distributions of positive and negative responses to those of the DMV plot and explore manually various parameter constellations. Such a comparison will be particularly useful for determining RT outliers, which are subject to an ongoing debate of how to be handled (cf. Grasman et al., 2009; Ratcliff, 1978; Vandekerckhove & Tuerlinckx, 2007).

Similarly, the DMV could serve as a validation tool for empirical data: for example, Schmiedek et al. (2007) used the DM parameters estimated from their data to simulate a DM perfectly in line with these estimates and compared statistics derived from the simulated data to the respective empirical counterparts (Schmiedek et al., 2007, p. 424, Fig. 3). With the DMV, they had the entire shape of the RT distributions at their disposal, rather than only statistics.

Most diffusion model parameter estimation routines allow for fixing of parameters and estimating the remaining ones (e.g., $z=0.5$ or variability parameters are set to zero). Comparing the observed with DMV-generated distributions may help determine a sensible choice of model parameter restrictions (requiring validation by independent data, of course). For example, in “Parameter variability/variability parameters”, we referred to studies arguing in favor of fixing the variability parameters. Exploring the effect of these parameters with the DMV reveals that they have rather moderate impact upon the RT densities compared to those of the four main parameters. This supports—at least to some extent—the critical voices regarding the variability parameters.

## Conclusions and outlook

Although the diffusion model was introduced quite a while ago in the late seventies, it was only scarcely applied during the early years of its existence. But within approximately the last decade, a number of easy-to-use programs have appeared and a steadily growing number of studies have been applying the DM. For this growing community, the DMV will likely serve as a useful tool and might come in handy during lectures and presentations. The program is available at https://osf.io/4en3b/ or http://www.dmvis.at/.
